# Thinking chickens: a review of cognition, emotion, and behavior in the domestic chicken

**DOI:** 10.1007/s10071-016-1064-4

**Published:** 2017-01-02

**Authors:** Lori Marino

**Affiliations:** The Someone Project, The Kimmela Center for Animal Advocacy, 4100 Kanab Canyon Road, Kanab, UT 84741 USA

**Keywords:** Chicken cognition, *Gallus domesticus*, Sentience Intelligence, Social complexity, Communication

## Abstract

Domestic chickens are members of an order, Aves, which has been the focus of a revolution in our understanding of neuroanatomical, cognitive, and social complexity. At least some birds are now known to be on par with many mammals in terms of their level of intelligence, emotional sophistication, and social interaction. Yet, views of chickens have largely remained unrevised by this new evidence. In this paper, I examine the peer-reviewed scientific data on the leading edge of cognition, emotions, personality, and sociality in chickens, exploring such areas as self-awareness, cognitive bias, social learning and self-control, and comparing their abilities in these areas with other birds and other vertebrates, particularly mammals. My overall conclusion is that chickens are just as cognitively, emotionally and socially complex as most other birds and mammals in many areas, and that there is a need for further noninvasive comparative behavioral research with chickens as well as a re-framing of current views about their intelligence.

## Introduction

When asked to think of an example of a bird, most people do not think of chickens (*Gallus gallus domesticus*) (Malt and Smith 1984). And when people see photographs of domestic chickens behaving like other birds (e.g., roosting in tree tops), it is often cause for surprise and amusement. Why? With over 19 billion worldwide, chickens are the most abundant of all domesticated animals (UN Food and Agricultural Organisation 2011), so this perception of chickens is not due to unfamiliarity with them per se. Rather, the answer may lie with the context in which we usually encounter them and how their use interacts with perceptions of their intelligence.

Unlike many other birds, chickens are categorized as a commodity, devoid of authenticity as a real animal with an evolutionary history and phylogenetic context. Thus, arguably, perceptions of chickens shape their use as commodities which, in turn, then reinforces those original perceptions. Animals are typically classified according to the kinds of attributes they possess (Mervis and Rosch 1981), and the contexts in which we usually encounter animals shape our views of how representative we think they are of a more general category (Malt and Smith 1984). When asked to rate the typicality of chickens as a member of the more general category of birds, raters usually give chickens a low score indicating that they are not considered typical birds (Malt and Smith 1984). Therefore, even considerations of birds in general may not apply very well to chickens.

And while many factors are involved in determining attitudes toward other animals, a number of studies have shown that belief in sentience or “mind” is a strong predictor of attitudes toward different types of animal use (Hills 1995; Knight and Barnett 2008; Knight et al. 2004; Phillips and McCulloch 2005). Chickens are misperceived as lacking most of the psychological characteristics we recognize in other intelligent animals and are typically thought of as possessing a low level of intelligence compared with other animals (Eddy et al. 1993; Nakajima et al. 2002; Phillips and McCulloch 2005).

Indeed, the very idea of chicken *psychology* is strange to most people. A recent study showed that when college students were given the opportunity to learn about and personally train chickens (using positive reinforcement), their attitudes shifted in a more informed and positive direction. Student perceptions of chicken intelligence were assessed pre- and post-training. Relative to their initial perceptions of chickens as slow learners, the students’ attitudes shifted to viewing them as intelligent and emotional animals with individual personalities. Interestingly, even pre-training, most students agreed that chickens could feel hunger, pain, and fear, but were less likely to believe chickens could feel more complex emotions, such as boredom, frustration, and happiness. However, boredom, frustration, and happiness were the emotional states with the greatest shifts in student attitudes post-training (Hazel et al. 2015).

The scientific literature on chicken cognition and behavior is relatively sparse in many areas, and dominated by applied themes, artificial settings, and methodologies relating to their “management” as a food source. In other studies, their welfare is ultimately related to productivity. Far less numerous are studies of chickens on their own terms—as birds, within an evolutionary and comparative framework. But even basic comparative studies of birds have been limited by concentrating almost exclusively on associative learning, discrimination, and adaptive specializations (such as seed caching), while interest in the evolution of complex intelligence has been focused mostly on primates, dolphins, elephants, and only certain birds, such as corvids (crows) and psittacines (parrots) (Emery 2006). Although it is beyond the scope of this paper to explore why this might be, arguably even the scientific community has been influenced by public perceptions of chickens as cognitively simple. Cognitive differences among species do indeed exist, but the fact that studies of very basic associative processes tend to focus on pigeons (*Columba livia*) and chickens (two species who are considered quite atypical as birds and not extremely favored), while studies of more complex cognitive processes, including language-like capacities and tool use, involve corvids and parrots, may have so far precluded chickens from demonstrating more complex cognition. As will be demonstrated in this paper, chicken intelligence appears to have been underestimated and overshadowed by other avian groups. This asymmetry in the literature is likely a reflection of, as well as a contributor to, the disconnect scientists and the public have between chickens as commodities and who they actually are as individuals.

But chickens have much in common with other avian species. Now, more than ever, this simple realization has a special relevance because of the recent transformation in our scientific knowledge of birds in general. In the past few years, numerous studies have shown that there is no “bright line” between “avian” and “mammalian” intelligence and complexity; complex intelligence is found in both birds, mammals, and also fish (Brown 2015; Butler 2008; Emery 2006). Likewise, the brains of birds have historically been viewed as simpler and more primitive than those of mammals. However, that assumption about avian brains has now been overturned by more recent studies showing that there are many functional similarities in the brains of birds and mammals, allowing for similar cognitive abilities. In particular, the avian forebrain (the part of the brain involved in problem-solving and other higher-order cognitive capacities) is actually derived from the same neuroanatomical substrate as the mammalian forebrain, providing more potential evidence for similar cognitive capacities in the two groups (Jarvis et al. 2005).

In this paper, I review the evidence from peer-reviewed applied and basic comparative studies of chicken cognition, emotion, and sociality. I place a special focus on more complex capacities which appear to be at the leading edge of intelligence in birds and other animals and only review some of the more fundamental perceptual and cognitive abilities in order to understand the mechanisms underlying these more complex capacities. A recent book on the behavioral biology of chickens by Nicol (2015) is recommended for a much more comprehensive and wide-ranging description of studies of chicken cognition and behavior.

The purpose of this paper is twofold: first, to gain a better understanding of the minds of chickens from the best scientific literature, separating fact from fiction; two, to identify compelling areas for future noninvasive research. Moreover, as with any taxonomic group, species-specific factors, such as evolutionary history and sensory abilities, need to be taken into account in order to interpret findings on cognition, emotion, sociality, and other characteristics and to make better informed comparisons across taxa. Therefore, what follows is a brief description of evolution, phylogeny, and domestication, as well as sensory systems, in chickens.

## Evolution, phylogeny, and domestication

Domestic chickens descended from red jungle fowl (*Gallus gallus*). They are considered a subspecies of their wild counterparts, who inhabit field edges, groves, and scrubland in India and southeast Asia (Al-Nasser et al. 2007). The domestication of the red jungle fowl was well established by 8000 years ago (West and Zhou 1988), but molecular studies suggest it could have begun as early as 58,000 years ago (Sawai et al. 2010).

Despite their long history of domestication, domestic chickens remain similar to their wild counterparts despite the recent very intense breeding and genetic manipulation directed toward production traits such as egg laying and growth (Rauw et al. 1998; Appleby et al. 2004). There is no evidence, for instance, that the cognitive or perceptual abilities of domestic chickens have been substantially altered by domestication. It is interesting to note that most animals domesticated for food, such as pigs and chickens, are behaviorally and cognitively quite similar to their ancestors and wild counterparts as they are mainly selected on physical characteristics like rate of growth, fecundity, percentage of body fat, etc. (Held et al. 2009). This stands in contrast to the case of dogs and wolves, who, of course, share a number of characteristics with each other but, because dogs were selected as companions, are also distinctly different on several key cognitive and behavioral dimensions (Udell et al. 2010). The implications for differential welfare for dogs versus chickens (and other farmed animals) in a “domesticated” setting are evident.

Social groups of jungle fowl and wild or free-ranging domestic chickens usually consist of one dominant male and one dominant female, subordinates of both sexes, and chicks, all occupying a home range during the breeding season (Appleby et al. 2004). Within their home range, they have regular roosting sites, including high up in the branches of trees (Appleby et al. 2004). Diet is highly varied and ranges from berries and seeds to insects and small vertebrates (Savory et al. 1978). Interestingly, despite the fact that domestication tends to make most animals less aggressive toward potential predators, some breeds of domestic chickens are more aggressive than jungle fowl (Väisänen et al. 2005).

## Sensory abilities

Chickens are sensitive to touch, and their skin contains numerous kinds of receptors for temperature, pressure, and pain. The beak of the chicken, as in all birds, is a complex sensory organ with numerous nerve endings. The beak not only serves to grasp and manipulate food items, but is also used to manipulate non-food objects in nesting and exploration, drinking, and preening. It is also used as a weapon in defensive and aggressive encounters. At the end of the beak is a specialized cluster of highly sensitive mechanoreceptors, called the bill tip organ, which allows chickens to make fine tactile discriminations (Gentle and Breward 1986). Needless to say, damage to the beak is intensely painful, as partially debeaked chickens show a significant increase in guarding behavior, i.e., tucking the bill under the wing, and diminished use of the bill for pecking and preening after the procedure. These pain-related behaviors may continue for months (Duncan et al. 1989; Gentle et al. 1990, 1991).

Chickens, like most birds, depend highly on well-developed visual abilities which allow them to focus close-up and far away at the same time in different parts of their visual field (Dawkins 1995; Dawkins and Woodington 1997), and see a broader range of colors than humans (Ham and Osorio 2007). Chickens can detect both low- and high-frequency sound at a variety of pressure levels. Their adeptness with low-frequency sound may include a capacity to detect sounds that humans cannot hear (infra-sound below 20 Hz) (Gleich and Langermann 2011). Chickens also possess well-developed senses of smell and taste (Jones and Roper 1997). Finally, like some other birds, chickens (though not all breeds) possess the ability to detect and orient to magnetic fields (Freire et al. 2008). All of these capacities come into play when assessing their cognitive capacities.

## Research methods

This paper presents a summary of cognitive, emotional, personality, and social characteristics of domestic chickens, built from a comprehensive review of the scientific literature. I first conducted a search on the Web of Science Core Collection using terms relevant to intelligence, cognition, and behavior and followed up with online Google-based direct searches through all of the major peer-reviewed journals (Table 1) using similar terms as well as key terms from existing papers (e.g., intelligence, cognition, behavior, learning, memory, sociality, self-awareness, etc.). I also used more specific search terms in Web of Science, e.g., time perception, perspective-taking, etc., within these broader categories when necessary. Additionally, I used these terms to search on ScienceDaily for relevant news items and the peer-reviewed papers they described. I also conducted a complete search of the Web sites of the major authors in these fields for all of their relevant projects. Finally, I searched the reference section of each paper to find additional papers in additional miscellaneous journals (not listed in Table 1) and ensured that the overall search was comprehensive. I included books, book chapters, and dissertation theses, as well as both empirical and review papers (which provided further description and interpretation of the empirical data). Both the basic comparative psychology literature and the applied literature were included. No time restrictions were placed on articles for inclusion, but priority was given to more recent papers when appropriate. The reference section of the present paper shows the full breadth of the sources consulted.

**Table 1:** List of the major peer-reviewed journals searched

Animal Behaviour
Animal Cognition
Animal Learning and Behavior
Animal Welfare
Anthrozoos
Applied Animal Behavior Science
Behaviour
Behavioural Processes
Current Biology
International Journal of Comparative Psychology
Journal of Comparative Psychology
Nature
Neuroscience and Biobehavioral Reviews
Physiology and Behavior
Public Library of Science—PloS One and Biology Science

## Visual cognition and spatial orientation

There is a deep literature on visual cognition and spatial orientation in chickens (including young chicks) that demonstrates they are capable of such visual feats as completion of visual occlusion, biological motion perception, and object and spatial (even geometric) representations. One of the cognitive capacities most extensively explored in this domain is object permanence, that is, the ability to understand that something exists even when out of sight. Object permanence unfolds in six developmental steps beginning, in Stage one, with a lack of understanding that hidden objects still exist and, in Stage two the ability to visually track the movement of an object. Stages three and four are reached when the subject actively retrieves a partially hidden and fully hidden object, respectively. Stages five and six are defined as the ability to track the location of a hidden object after several visible displacements and infer its location after several invisible displacements, respectively (Piaget 1953). Human babies typically achieve the last stage at about age 2 years (Piaget 1964).

Object permanence has been studied extensively in many nonhuman animals, who show a range of capacities within this paradigm. The literature on this phenomenon in other animals is too extensive to cite here but suffice to say that many animals, such as great apes, monkeys, cats, dogs, and birds, demonstrate various levels of sophistication in object permanence, with many achieving competence in the final of the six stages (see Gomez 2005, for a review). I will turn to an examination of object permanence in chickens in the following sections on partly and completely occluded objects.

### Recognizing partly occluded objects

A number of birds are capable of reaching for partly occluded objects (amodal completion), the equivalent of Stage 3 object permanence. To name a few, parrots (*Psittacus erithacus*), parakeets (*Melopsittacus undulates* and *Cyanoramphus auriceps*), macaws (*Ara maracana*) (Funk 1996; Pepperberg and Funk 1990), mynahs (*Gracula religiosa*) (Plowright et al. 1998), magpies (*Pica pica*) (Pollok et al. 2000), Eurasian jays (*Garrulus glandarius*) (Zucca et al. 2007),and carrion crows (*Corvus corone*) (Hoffman et al. 2011) pass these tests (as well as more advanced stages of object permanence, including, in some, Stage six competence) easily. Pigeons (*Columbia livia*), on the other hand, seem to lose interest in food when it is placed behind an opaque screen (Plowright et al. 1998).

Most studies of the ability to recognize partly hidden objects in chickens have employed a paradigm that involves imprinting just-hatched chicks onto a geometric shape, such as a red triangle, and testing them later to determine which of two versions (a partly occluded triangle or a triangle with a piece missing) they prefer (choose to be near). Chicks choose the partially occluded triangle (Regolin and Vallortigara 1995), just as humans do. The reasoning behind this finding is that the chicks, like humans and some other animals, are “filling in” the occluded part of the triangle and, therefore, perceiving it as the whole object upon which they are imprinted. Some studies, using different stimuli and protocols, have suggested the same general conclusion for both chicks (Lea et al. 1996) and adult hens (Forkman 1998). However, it isn’t clear that the numerous methods used to assess amodal completion in chicks and in adult hens are similar enough to reveal actual cognitive similarities between the two age groups (Nakamura et al. 2010). Indeed, even humans have difficulty with amodal completion under certain circumstances that pigeons and chickens do not (Nakamura et al. 2014). These findings caution that there is a great deal of heterogeneity within even one region of cognitive abilities, in this case, amodal completion, across and within species.

One of the ways the ability to represent partly hidden objects can be further tested is by determining whether an animal sees subjective or illusory contours, i.e., parts of a whole shape only “suggested” by occlusion. A number of mammalian species perceive subjective contours, e.g., cats (Bravo et al. 1988) and monkeys (*Macaca mulatta*) (Peterhans and von der Heydt 1989). Birds, e.g., barn owls (*Tuto alba*) (Nieder and Wagner 1999), fish, e.g., redtail splitfin (*Xenotoca eiseni*) (Sovrano and Bisazza 2009) and goldfish (*Carrassius auratus*) (Wyzisk and Neumeyer 2007) and even invertebrates, e.g., bees (*Apis mellifera*) (van Hateren et al. 1990; Nieder 2002, for a review) perceive subjective or illusory contours. Two-week-old chicks also perceive subjective contours (Zanforlin 1981). Therefore, these perceptual abilities are rather pervasive, although not universal, in the animal kingdom. Interesting questions arise when considering the depth and abstractness of processing of such visual percepts across taxa.

### Recognizing completely occluded objects

Tests of Stage 4 object permanence are similar to those of Stage 3 except objects are completely hidden. Chicks as young as two days old master some, but not all, aspects of Stage 4 object permanence (see Regolin et al. 1994; Vallortigara and Regolin 2002; see also Campbell 1988, for similar evidence in adult hens). For instance, although chicks do have an object concept that maintains a representation of the object in the absence of direct sensory cues, it seems that they are not as easily able to predict the resting position of an imprinted ball from its direction of movement prior to occlusion (Freire and Nicol 1997, 1999). However, chicks are able to choose the correct screen when the goal-object is a “social” partner (i.e., a red ball on which they had been imprinted) (Chiandetti and Vallortigara 2011). Moreover, chicks also appear to make use of the directional cue provided by the movement of the prey when they are tested in the presence of a cage-mate (Regolin et al. 1995). These studies point to the interesting fact that chickens, like other social animals, often perform better on tasks which tap into their social propensities. Consistent with this idea is the fact that chicks also have a preference for approaching a point-light stimulus moving in a more biologically natural way, i.e., like a walking hen, than the same lights randomly moving, as they align their bodies in the same direction of the apparent movement of the “hen” (Regolin et al. 2000).

In summary, the evidence for Stage 3 object permanence in chickens is fairly strong but more work needs to be done to elucidate the mechanisms behind completion of these tasks in very young chicks versus adult hens. Young chicks do show some capacities related to Stage 4 object permanence, but these abilities seem to be limited to tasks with stimuli that resemble natural social situations.

## Numerical abilities

In the last few decades, there has been a growing scientific literature on the numerical competencies of nonhuman animals. While there is still much debate about what these abilities mean in nonhuman animals (and even young human children), they are arguably related to mental representation of some kind (Dehaene et al. 1999). At the most basic level is the ability to discriminate between two or more sets of objects that are different on the basis of number of objects in each set, e.g., “more than…” or “fewer than…”. Several species show preferences for the larger amount when deciding between two quantities, including chimpanzees (*Pan troglodytes*) (Boysen et al. 2001), orangutans (*Pongo pygmaeus*) (Call 2000), rhesus macaques (*Macaca mulatta*) (Hauser et al. 2000), bottlenose dolphins (*Tursiops truncatus*) (Jaakkola et al. 2005; Kilian et al. 2003), lions (McComb and Packer Cm Pusey 1994), elephants (*Elephas maximus*) (Irie-Sugimoto et al. 2009), and horses (*Equus caballus*) (Uller and Lewis 2009), among others.

A more sophisticated capacity closer to a real number concept is ordinality, the ability to place quantities in a series. Competence in ordinality is found in several species, including many of those above, e.g., chimpanzees (Boysen and Bernston 1990), rhesus macaques (Brannon and Terrace 2000), and also pigeons (Brannon et al. 2001), crows (Smirnova et al. 2000) and African grey parrots (Pepperberg 1994, 2006).

Experiments with newly hatched domestic chicks (Rugani et al. 2008, 2010; Vallortigara et al. 2010) show that they are capable of discriminating quantities and a simple form of ordinality. Chicks were reared with five identical objects (small balls) on which they imprinted. On days 3 or 4, chicks underwent free-choice tests in which two sets containing three and two balls disappeared (either simultaneously or one by one), each behind one of two opaque identical screens. Chicks spontaneously inspected the screen occluding the larger set. In the next experiment, after the initial disappearance of the two sets, some of the objects were visibly transferred, one by one, from one screen to the other. Thus, computation of a series of subsequent additions or subtractions of elements that appeared and disappeared, one by one, was needed in order to perform the task successfully. Chicks chose the screen hiding the larger number of elements at the end of the event, irrespective of the directional cues provided by the initial and final displacements. These experiments also showed that chicks have a sense of a “mental number line” indicative of ordinality (Rugani et al. 2007).

Rugani et al. (2009) demonstrated that five-day-old domestic chicks are able to perform arithmetic operations to a total of five objects (Rugani et al. 2009). When they were presented with two sets of objects of different quantities disappearing behind two screens, they were able to successfully track which screen hid the larger number by apparently performing simple addition and subtraction. Finally, in a compelling demonstration of shared cognitive propensities in chicks and humans, Rugani et al. (2015) showed that chicks always associate the smaller of two quantities with the left, rather than right, spatial location. The authors suggest that, due to similar neural architecture, the chicks, like many other species, have a shared predisposition to map numbers onto geometrical space in a similar way.

It is clear that chickens, as a species, share a number of sophisticated cognitive capacities with other animals. However, because these studies depend heavily upon imprinting paradigms they are weighted toward studies with very young animals. These early-emerging core abilities do not exclude learning, particularly in a social context, as an important driver of chicken cognition any more than it does in humans with similar precocial capacities. But there is a paucity of information about how these abilities play out developmentally into adulthood in chickens, and more information is urgently needed about this process to gain better insight into what these capacities mean for cognitive complexity in a comparative context.

## Time perception/anticipation of future events

An area of longstanding interest in comparative cognition is time perception, i.e., the ability to detect the passage of time. In general, time perception has to do with the question of whether other animals live entirely in the present or can anticipate a future.

Basic time perception is considered by many scientists to be requisite for the more sophisticated process of mental time travel—the conscious ability to mentally represent the past and plan for the future. The ability to travel backwards in time and recollect specific past events is called episodic memory. It has been argued that episodic memory is tied to mental time travel (Dere et al. 2006). Arguably, therefore, when coupled with an episodic memory system, time perception becomes evidence for an autobiographical sense of self in the past, present, and future.

### Perception of time intervals

Many animals have a sense of time duration, which helps them to know the time of day and predict when events will occur (Gallistel 1994; Richelle and Lejeune 1980). Domestic pigs (*Sus scrofa domesticus*) for instance, show a capacity for temporal response differentiation (Ferguson et al. 2009) and distinguishing between short versus long time intervals (Spinka et al. 1998). Furthermore, they are able to anticipate future negative or positive events (Imfeld-Mueller et al. 2011).

Chimpanzees (*Pan troglodytes*) and other great apes show sophisticated abilities in the time perception realm, as they are able to prepare themselves for future actions (e.g., tool use: Beran et al. 2004; Osvath and Osvath 2008) even as much as 14 h in advance (Mulcahy and Call 2006). They also demonstrate a capacity for episodic memory. They can remember highly specific contextual elements; that is, the *what, where, and when* of events when an hour or even two weeks have passed (Martin-Ordas et al. 2010, 2013). Bottlenose dolphins also show robust evidence of episodic memory in complex tasks requiring them to directly access memories of behaviors they have performed previously (Mercado et al. 1998).

At the simplest level, studies of time perception in birds have shown that a number of avian species, e.g., pigeons (Roberts et al. 1989) and black-capped chickadees (*Parus atricapillus*) (Brodbeck et al. 1998), are able to estimate short time intervals of up to 60 s. This has been demonstrated using operant conditioning techniques in which the pattern of peck responses indicates the bird’s ability to anticipate an upcoming food reward. However, these and other bird species have shown temporal abilities that go beyond these findings when given the opportunity. For instance, one study with pigeons showed they were capable of judging intervals of up to 8 min (Zeiler and Powell 1994). Western scrub jays (*Aphelocoma californica*) make provisions in advance for a future need, both by preferentially caching food in a place where they have learned that they will be hungry the next morning, and by differentially storing particular food items in a place in which that type of food will not be available the next morning (Raby et al. 2007).

In the only study directly testing time perception in chickens, five thirty-week old hens were able to predict, approximately, a 6-min interval when given a reliable predictive visual signal (Taylor et al. 2002). The hens were required to peck a computer-controlled touch screen that delivered a food reward upon the first peck after 6 min. The hens showed they were capable of estimating the time interval by showing a pattern of increased pecking frequency around the 6-min mark. As good as the chicken’s performance was, it should be noted that they were able to achieve this performance within a highly artificial setting. Almost certainly, a more naturalistic setting would allow the chickens’ temporal abilities to be more easily demonstrated, as all animals, including birds, depend upon the appropriate environmental context for the full expression of their behavior.

In another study which tapped into time perception through an anticipatory emotional response, laying hens were taught to discriminate three sounds which signaled either a positive outcome (food reward), a negative outcome (a squirt from a water gun) or a neutral outcome (nothing) after a 15-s delay. The hens showed differential emotional responses to the different sounds suggesting that they were able to anticipate a future outcome (Zimmerman et al. 2011). More details about the birds’ emotional responses can be found in the section on Emotions below.

### Episodic memory

Studies of episodic memory provide a window into the question of whether other animals remember personal experiences, i.e., possess episodic memory. Episodic memory, a component of declarative memory, is tied to whether an individual experiences life autobiographically (autonoetic consciousness). Tulving (2005) defined episodic memory in terms of its subjective experience. Moreover, the demonstration of episodic memory in other animals has been argued to be probative of autonoetic conscious experience, as it relies upon distinctive personal memories (Dere et al. 2006; Eichenbaum et al. 2005; Martin-Ordas et al. 2013).

In addition to many mammals, including great apes (Martin-Ordas et al. 2013; Schwartz et al. 2005), a number of bird species demonstrate evidence for memory described as “episodic-like” (Clayton and Dickinson 1998). In a visual discrimination task which allowed for control over confounding variables, Zentall et al. (2001) found some evidence for episodic memory in White Carneaux pigeons. In this study, the pigeons were essentially asked the question: “Did you just peck or not?” and they remembered specific details which allowed them to “answer” this question with key pecks. In other studies, pigeons have demonstrated meta-knowledge about the behavior they just emitted, that is, knowledge about their own knowledge (Shimp 1982).

But in other studies, the evidence for metacognition is inconclusive (Iwasaki et al. 2013). Western scrub jays (*Aphelocoma coerulescens*) show evidence of episodic memory, i.e., the *what, where, and when* of food-caching episodes. Jays can remember when and where they cached a variety of foods that differ in the rate at which they decay, and retrieve those stored foods later in the appropriate order. They can update their memory of the contents of a cache depending upon whether they have previously visited the site. Furthermore, they can also remember where other birds cache their food, showing that they encode rich mental representations of caching events (Clayton et al. 2001, for a comprehensive review of these studies). Although there has been some debate about whether these findings represent episodic memory or other forms of associative learning (Suddendorf and Corballis 2007), these criticisms have been disputed (Raby et al. 2007). Clearly, some very interesting complex cognitive processes are coming into play in these food-caching behaviors.

In a more direct test of metacognition in scrub jays, the birds were required to allocate a proportion of time looking into two peepholes in order to see food being hidden in either of two compartments, one where observing the hiding location was necessary to later relocate the food, and another where food could easily be found without watching. The jays first separately experienced the consequences of possessing information in each compartment and subsequently, once given a choice, made more looks and spent more time looking into the compartment where information was necessary than into the compartment where it was unnecessary. Thus, the jays showed that they not only can differentiate sources of information according to their potential value but they can collect information needed to solve a *future* problem (Watanabe et al. 2013).

As mentioned above, the presence of episodic memory in chickens might be inferred from findings like the ones described above on time perception and anticipation, which probe capacities that are correlated with episodic memory. But there are other ways to more directly investigate the presence of episodic memory in chickens. Studies of memory using a matching-to-sample paradigm may reveal episodic-like memory components because they require the subject to “declare” the characteristics of a stimulus they have kept in memory. Hens can successfully complete these tasks, but the delays used are typically very short (on the order of seconds, see Foster et al. 1995). In studies of Stage 4 object permanence like those described above, episodic memory can be tested by imposing a delayed response procedure that requires maintaining a memory of a specific event over a longer period of time than just a few seconds. Chickens are able to remember the trajectory of a hidden ball for up to 180 s if they could see the ball moving and up to 1 min if the displacement of the ball was invisible to them (Vallortigara et al. 1998). In other words, they did as well as most primates (Wu et al. 1986) under similar conditions.

In other studies, five-day-old chicks were fed with two plates, each with a different kind of food. The food was devalued by pre-feeding with one of the food types, thus decreasing the novelty and incentive for that food type compared with the other. When tested later (on the order of a few minutes), the checks went to the location where they had previously found food (Cozzutti and Vallortigara, 2001). Similar results have been found for hens (Forkman 2000) showing that chicks and adult chickens are capable of remembering the “where” and “what” components of information about food.

### Self-control

Self-control can be broadly defined as the ability to resist immediate gratification for a later benefit. It may be associated with planning for the future because foreplanning requires not only mental time travel, but the ability to inhibit or delay a response until later. However, the relationship between self-control and planning for the future is still in need of clarification in many studies.

Self-control may also be associated with the development of self-awareness (Genty et al. 2004) and autonomy—the ability to think about and choose future outcomes. Self-control is typically not reliably demonstrated in human children until they are at least 4 years of age (Mischel et al. 1989). Self-control is generally assessed in humans and other animals by determining whether they can delay obtaining a small reward for a larger reward later. Thus, these tests are prospective timing tasks requiring prediction of an outcome in the future based on experience in the past. Many mammals show self-control under these circumstances, including rats (*Rattus norvegicus*) (e.g., Chelonis et al. 1998; Flaherty and Checke 1982), and primates, such as lemurs (*Eulemur fulvus* and *E. macaco*) (Genty et al. 2004), rhesus monkeys (*Macaca mulatta*) (Beran et al. 2004), chimpanzees and orangutans (*Pongo pygmaeus*) (Beran 2002; Osvath and Osvath 2008).

A number of avian species demonstrate self-control in experimental situations, including pigeons (e.g., Logue et al. 1985; Mazur 2000), black-capped chickadees (Feeney et al. 2009, 2011), and, in a similar paradigm to that used with primates, the carrion crow (*Corvus corone*) and the common raven (*Corvus corax*) (Dufour et al. 2011).

Domestic chickens, too, show the capacity for self-control in an experimental setting. In a situation where they are given a choice between a 2-s delay followed by access to food for 3 s or a 6-s delay followed by access for 22 s (a veritable jackpot), hens held out for the larger reward, demonstrating rational discrimination between different future outcomes while employing self-control to optimize those outcomes (Abeyesinghe et al. 2005). Given the promising results of this study, more exploration of the cognitive basis of self-control in chickens is indicated.

## Reasoning and logical inference

The ability to reason and apply logic is a hallmark of intelligence in humans and nonhumans alike. Perhaps the kind of logical reasoning most explored in animals other than humans is a form of syllogism called transitive inference. Transitive inference is a type of deductive reasoning that allows one to derive a relation between items that have not been explicitly compared before. In a general form, it is the ability to deduce that if Item B is larger than Item C and Item C is larger than Item D, then Item B must be larger than Item D (Lazareva 2012). This form of inference has been described as a cognitive developmental milestone unique to humans who are at least 7 years of age and in the concrete operational stage of development (Piaget 1928).

However, there is now evidence for transitive inference in a wide range of nonhuman animals, including chimpanzees, various species of monkeys, rats, and several avian species (see Vasconcelos 2008, for a review of this literature). Chickens have also demonstrated this capacity (Hogue et al. 1996). When hens are placed together for the first time, they set up a dominance hierarchy—a pecking order. Dominant hens defeat subordinates by pecking at them, jumping on them, or clawing them. Subordinates show submission by crouching or trying to get away. In this study, hens were placed with others in dyads and triads to determine how hens use information about the relationships among others to assess their own position in the pecking order when confronting a new individual. In one condition, hens witnessed a familiar dominant individual being defeated by a stranger and then they were introduced to the stranger. In another condition, the hens observed a familiar dominant hen defeat a stranger. In a third condition, the subjects witnessed two strangers establishing a dominance relationship before being introduced to their prior dominant and to a stranger the former had just defeated.

Subjects in the first condition, after seeing a known dominant individual being defeated by the stranger, did not challenge the stranger when confronted. Their actions indicated they understood that if this stranger can defeat someone who can defeat *them*, then they are not going to defeat that stranger. In the second condition, the hens attacked the stranger half of the time, indicating that they understood they had some chance of defeating her. In the third condition, the proportion of times the hens first approached the stranger matched whether they saw the stranger being defeated by the dominant hen or not. These results, altogether, indicate that hens can gain useful information about their status in the dominance hierarchy before actually engaging another hen by observing how that hen interacts with a “known entity” (the prior dominant hen). The results of this study are consistent with the idea that the hens were making self-assessments based upon the logic of transitive inference. They also show that, while simple processes can sometimes be the basis of complex-looking behavioral phenomena, sophisticated logical reasoning may underlie what is perceived to be a rather simple behavior—the pecking order.

There is still some discussion in the literature about the fundamental nature of transitive inference in nonhuman animals (Vasconcelos 2008). Nevertheless, social animals, including chickens, seem capable of employing some level of logical reasoning in important adaptive domains. As discussed below, this ability supports the emergence of complex social relationships in many nonhuman animals.

## Self-awareness

Self-awareness is subjective awareness of one’s identity, one’s body, and one’s thoughts through time, distinguished from others. In other words: a sense of “I.” The question of self-awareness in other animals appears to be on the extreme cutting edge of our ability to assess who they are to themselves. Self-awareness has been associated with a variety of related concepts, including phenomenal consciousness, self-consciousness, metacognition, and autonoetic consciousness. All of these terms converge upon the fundamental capacity to be aware of one’s independent existence in the physical and/or psychological domain. Importantly, the concept of self-awareness is likely to be multidimensional and, given the developmental evidence, best thought of as a continuum of awareness (Marino 2010). There are two studies that bear on the question of self-awareness in chickens: self-control and self-assessment.

### Self-control

As discussed above, chickens show self-control in experimental situations (Abeyesinghe et al. 2005) which require them to forgo an immediate reward for a later larger reward. Some authors have argued that self-control is indicative of self-awareness (Genty et al. 2004), as it tends to emerge reliably in humans at around the age of four, when other cognitive capacities related to self-awareness (e.g., mirror self-recognition) have either developed or are developing (Mischel et al. 1989). Although self-control is not direct evidence of all forms of self-awareness, it may be an important indicator of a sense of self at some level (but see Ainslie 1974; Rachlin and Green 1972, for other interpretations). It has been hypothesized that self-control depends upon the presence of episodic memory, and implying some capacity to mentally work through different scenarios for the future and choose the one providing the best option (e.g., the biggest reward) (Boyer 2008; Osvath and Osvath 2008). Thus, the presence of self-control over time in chickens may indicate a cognitive capacity on a continuum of complexity with foreplanning and mental time travel.

Moreover, self-control may be related to self-agency, the subjective awareness that one is initiating, executing, and controlling one’s own volitional action in the world (Kaneko and Tomonaga 2011). However, no direct tests of self-agency in chickens have been conducted, and this concept remains essentially unexplored, making it an excellent option for further study.

### Self-assessment

Another component of a sense of self is the ability to compare oneself to others as a distinct entity. Among birds, Greylag geese and pinyon jays (*Gymnorhinus cyanocephalus*) can infer their own social status by observing unfamiliar individuals interacting with familiar birds (Weiß et al. 2010; Paz-y-Mino et al. 2004) Chickens can apply logical inference to social situations as well. As Hogue et al. (1996) showed in their study of transitive inference, chickens can observe the interactions of an individual of known status with an unknown individual and infer their own status in the social hierarchy relative to the unknown individual and respond appropriately (e.g., dominantly or submissively) in future interactions. These studies show that in socially complex birds, such as chickens, logical inference is likely important for navigating their social landscape.

## Communication

Communication involves the transfer of information from one individual to another—a critical component of social complexity. The study of communication in animals involves characterizing its functionality, contexts, uses, structure, and complexity. There is still considerable debate in the animal communication literature about the nature of communication in other animals, including how it compares with human languages. Many theorists still have reservations about the depth and complexity of animal communication systems. These reservations are often based in the assumption that human language is entirely unique. Animal “signals,” in comparison, are said to be involuntary products of emotional states, lacking in intentionality, richness, and flexibility, without connection to cognition and thinking (e.g., Berwick et al. 2013; Lieberman 1994; Luria 1982; Premack 1975). Although just like humans, animals do sometimes communicate in nonlinguistic, involuntary affective displays; some animal communication is clearly cognitively complex, reflecting flexible mental representations. In fact, there is an abundance of evidence for complex, flexible, and rule-governed natural communication systems across a wide array of species (Slobodchikoff 2012).

Chicken communication consists of a large repertoire of at least 24 distinct vocalizations, as well as different visual displays (Collias 1987; Collias and Joos 1953). But the sophistication of chicken communication comes to the forefront when one examines how these vocalizations are used and the cognitive capacities they apparently rely upon.

### Referential communication

Referential communication involves signals (calls, displays, whistles, etc.) which convey information, i.e., refer to specific elements of the environment. What makes referential communication so interesting and complex is that it implies that the animals using it attach meaning to each signal in a way not unlike the way humans use words for objects and other entities in our world. In other words, referential communication has semanticity. It is generally studied by observing and recording a signal’s usage and then using playback recordings in an experimental manipulation to determine the actual meaning and use of the signal to the receivers. If a very tight correlation between the specific eliciting event and the receivers’ responses is found, the signals can be said to be referential, i.e., function to convey information about the content or nature of the event and, often, the appropriate response.

Referential communication stands in contrast to long-held assumptions that animal signals are only reflexive “stimulus-bound” responses, or contain only very low level information about affective state (e.g., aggression) or physical attributes of the caller (e.g., size). Referential communication shows that there are important cognitive components to animal communication requiring intentionality and mental representation. That is, referential communication serves to evoke mental representations of the eliciting event in the minds of the receivers (Evans 1997, 2002; Evans and Evans 2007).

Functionally referential communication has been identified in many mammal and bird species. Vervet monkeys were the first species found to have referential communication. They have acoustically distinct alarm calls corresponding to three different types of predators, each of which requires a different type of response on the part of the receivers (Cheney and Seyfarth 1990; Seyfarth et al. 1980; Struhsaker 1967). Referential communication is also found in ring-tailed lemurs (*Lemur catta*) (Macedonia 1990), chimpanzees (Slocombe and Zuberbühler 2005), Diana monkeys (*Cercopithecus diana*) (Zuberbühler 2000), bottlenose dolphins (Janik et al. 2006), black-tailed prairie dogs (*Cynomys ludovicianus)* (Frederiksen and Slobodchikoff 2007), and domestic dogs (Gaunet and Deputte, 2011; Miklósi et al. 2000; Polari et al. 2000) to name a few mammal species. Several species of birds also engage in referential communication, including ravens (Bugnyar et al. 2001) and chickadees (Templeton et al. 2005), among others.

Chickens, too, demonstrate considerable complexity in their use of referential communication. When shown computer-generated animations of natural predators, roosters emit distinctive alarm calls. For example, when shown aerial predators (e.g., a raptor flying overhead), they give one alarm call, and when shown a terrestrial predator (e.g., raccoon), they give another distinct alarm call (Evans et al. 1993a, b). The strongest alarm calls are made when a large, fast-moving hawk appears overhead (Evans et al. 1993a, b). The differential responses show specificity in their alarm calls. Likewise, receivers of these calls react to them in specific and appropriate ways, showing that the calls have the same meaning for all of the individuals in the group.

To add to the complexity of this behavior, males often employ risk compensation tactics which shape their communicative behavior when a predator appears (Kokolakis et al. 2010). For instance, a male is more likely to make an aerial alarm call when a female is present, which increases the chances of his mate and offspring surviving (Wilson and Evans 2008). There is considerable flexibility—and strategy—in alarm calling as well. By varying the composition and duration of the call, the male can still alert his social group while also confusing the predator about his exact location (Wood et al. 2000; Bayly and Evans 2003). For instance, a male will more likely sound an alarm if a subordinate is nearby, thereby giving the predator more than one target to hone in on (Kokolakis et al. 2010). Moreover, males give longer duration alarm calls (which are easier for prey to locate than shorter ones) when under cover of a tree or bush, suggesting that the rooster may have some understanding of the visual perspective of the aerial predator (see Perspective-taking and social manipulation below). These and other studies show that chickens are sensitive to “audience effects,” that is, their communication behavior is mediated by who is available to receive the call. For instance, males call far more often when a familiar conspecific is present than if he is alone or with a member of another species (Karakashian et al. 1988). Taken together, audience effects are consistent with the suggestion that communication in chickens is volitional and shaped by cognition and social awareness. However, much more research is needed to clarify the cognitive basis for the behaviors described above.

In addition to alarm calls, males also make food calls when they find a delectable tidbit. They combine these calls with rhythmic movements involving picking up and dropping the food morsel repeatedly—a signal called the tidbitting display. This referential display is loud and individually distinctive, broadcasting the identity of the caller to the whole group. This display is enmeshed in the complex social relationships among individuals in each group, as hens use it to determine which males will provide food and, thus, with whom they want to mate (Evans and Evans 1999; Pizzari 2003). Moreover, the vigor of the display is correlated with the quality of the food and the chances that a female will approach (Marler et al. 1986).

The years of experimental work on chicken communication show that it is vastly more complex than originally thought, suggesting the existence of cognitive awareness, flexibility, and even more sophisticated capacities such as perspective-taking and intentional or tactical deception (see the section below). As with other areas, chickens’ communication skills provide evidence for similarity with other highly intelligent complex social species, including primates.

## Social cognition and complexity

Social cognition is the use of cognitive skills (learning, memory, reasoning, problem solving, decision making, etc.) within the social domain, forming the basis for cognitive complexity and intelligence across a wide range of species. For many highly social animals, complex cognitive capacities are most clearly demonstrated when applied in a social setting, suggesting that many of these abilities evolved as adaptations to social living (Evans 2002). There is an abundance of empirical evidence showing a positive correlation between various high-level cognitive capacities and measures of social complexity in species as wide-ranging as domestic pigs (Marino and Colvin 2015, for a review), dogs (Bensky et al. 2013, for a review), primates (e.g., Dunbar 1998), dolphins and whales (Whitehead and Rendell 2015), and birds (Burish et al. 2004). These social cognitive capacities are important indicators of a flexible and dynamic intelligence and are intertwined with other dimensions of psychology, such as emotional responding and personality.

Chickens, like many other animals, demonstrate their cognitive complexity when placed in social situations requiring them to solve problems. Furthermore, chickens show even greater psychological complexity by flexibly, and often strategically, navigating a dynamic network of social relationships.

### Discriminating among individuals

The ability to discriminate among individuals forms the basis for social relationships, hierarchies, and reactions to familiar versus unfamiliar individuals. Individual discrimination is a prerequisite to the more complex capacity of true individual recognition, defined as a mental representation of an individual’s identifying characteristics. Thus, individual discrimination is a logical beginning for investigating a species’ general social recognition abilities.

The range of social species that can discriminate individuals in their social group is wide. Among mammals, dogs (Molnar et al. 2009), pigs (de Souza et al. 2006; McLeman et al. 2005), elephants (McComb et al. 2000), vervet monkeys (*Chlorocebus pygerythrus*) (Cheney and Seyfarth 1980), dolphins (Sayigh et al. 1999), macaques (*Macaca mulatta*) (Parr et al. 2000), chimpanzees (Parr et al. 2000), and numerous others have been shown to have this ability. The literature on vocal recognition in songbirds is well known and voluminous.

Visual recognition of conspecifics has also been demonstrated by birds, e.g., rooks, *Corvus frugilegus* (Bird and Emery 2008), pigeons (Nakamura et al. 2003), white-throated sparrows*, Zonotrichia albicollis* (Whitfield 1987), and budgerigars, *Melopsittacus undulatus* (Brown and Dooling 1992), to name a few. Some birds can also discriminate conspecifics on the basis of odor, e.g., Antarctic prions (*Pachyptila desolata*) (Bonadonna et al. 2007).

Chickens, too, show notable abilities to recognize individuals in their social group, as well as the ability to keep track of the group’s social hierarchy and the individuals within it (as discussed previously). Not only do chickens recognize who is and is not a member of their social group, but they differentiate individuals within their own group. Under various experimental conditions, domestic chickens have demonstrated the capacity to visually discriminate and recognize a large number of conspecifics presented live (Bradshaw 1991, 1992; D’Eath and Stone 1999) and in color slides (Bradshaw and Dawkins 1993; Ryan and Lea 1994).

### Perspective-taking and social manipulation

The ability to take the perspective of another individual is a complex cognitive capacity that allows an individual not only to respond to conspecifics, but also manipulate them. The most basic form of visual perspective-taking requires taking a viewpoint other than one’s own, sometimes using that information to one’s advantage. This capacity is often referred to as Machiavellian Intelligence (Whiten and Byrne 1997), defined as a kind of sociopolitical maneuvering involving deceit and manipulation of others’ mental states. It is considered a driver of the evolution of intelligence in primates, including humans (Humphrey 1976; Whiten and Byrne 1997). Perspective-taking has been associated with a number of other cognitive capacities, including self-awareness, theory of mind, intentional deception, and empathy in primates (Bulloch et al. 2008; de Waal 2008; Towner 2010, for a comprehensive review of these issues). A number of highly intelligent species have demonstrated well-developed capacities in the realm of conspecific perspective-taking, including chimpanzees (Krachun and Call 2009), dogs (Bräuer et al. 2013), pigs (Held et al. 2000, 2002) and, in the avian domain, Western scrub jays (Clayton et al. 2007). Here, too, domestic chickens show compelling abilities.

Returning to the tidbitting display, because the vocal and behavioral components of the display are redundant, a receiver of either one of the components will get the message indicating the presence of food. This dual-component nature of the tidbitting display is used by subordinate males to their advantage. Dominant males who hear subordinate males giving the tidbitting display will often attack and then displace the subordinate male. To minimize this occurrence, subordinates tend to omit the more conspicuous vocal components and restrict themselves to the movements of the visual display. However, when dominant males are distracted by something else, the subordinate adds back in the vocal component, which serves to attract females who are eavesdropping. This behavior suggests that the subordinate male is taking the perspective of the dominant male and using information about his attentional state to personal advantage (Smith et al. 2011).

Deception is another example of possible Machiavellian Intelligence in chickens. Males will sometimes make a food call in the absence of any food. This serves to attract females who, once near them, can be engaged and defended against other males (Gyger and Marler 1988). Of course, females develop counter-strategies and eventually stop responding to males who call too often in the absence of food (Evans 2002). These kinds of social strategies—deception and counter-strategies—are striking similar to the same kinds of complex behaviors identified in mammals, including primates.

### Social learning

One of the ways that social species take advantage of group living is through social (observational) learning—observing conspecifics’ behavior and its consequences in order to avoid time-consuming and sometimes hazardous “trial and error” learning. Social learning appears to be a form of deferred imitation (action learning) or emulation (results learning), serving as a mechanism for the transmission of learned behaviors over stretches of time, i.e., culture. But imitation and emulation are only two of a number of potential mechanisms for social learning (Zentall 2012), and careful experimentation is needed to differentiate among the many cognitive bases for social learning in other animals.

Many animals engage in social learning, including chimpanzees (e.g., Yamamoto et al. 2013), capuchin monkeys (*Cebus apella*) (Ottoni and Mannu 2001), and birds, such as ravens (Bugnyar and Kotrschal 2002) and quail (Koksal and Domjan 1998), to name a few. Chickens, too, engage in social learning to avoid the costs of direct learning (Nicol 2006). The use of syllogistic logic in determining the status of self and other in the social hierarchy is a strong example of observational learning in chickens (Hogue et al. 1996). Moreover, naïve hens who watched a trained hen perform a task were able to perform that task correctly more often than those who watched another naïve hen (Nicol and Pope 1992, 1994). Among conspecifics, the identity and social status of the demonstrator is important, as chickens learn from dominant individuals more readily than subordinates (Nicol and Pope 1999). Moreover, this effect is not based upon the fact that dominant individuals perform the task better than subordinates (Nicol and Pope 1999). Rather, it seems to be based upon the fact that more attention is paid to dominant individuals than others in the group. Therefore, in chickens, as in other animals, social factors mediate learning factors in a complex way.

## Emotion

Emotions are comprised of behavioral, neurophysiological, cognitive, and conscious subjective processes (Mendl and Paul 2004; Paul et al. 2005). Cognition can modulate emotional responses and visa versa (Mendl et al. 2009; Paul et al. 2005). Many studies of emotions in other animals, including chickens, refer instead to “affective state” or “core affect” (Fraser et al. 1997). “Affect” typically is discussed as either a pleasurable or displeasurable state (otherwise known as valence), coupled with some degree of intensity or arousal (Barrett 2006). The relationship between affect and emotion is complex, containing a number of components still widely debated on a theoretical level (Barrett 2012). Emotions are considered more cognitively based than affect, but are shaped by affect. It may be argued that some authors use the term “affect” instead of “emotion” to be conservative about claiming other animals have complex psychological states. Nevertheless, there is a large body of literature demonstrating complex emotions in other animals, including chickens.

For a long time, the study of emotions in other animals, including chickens, was focused exclusively on negative emotions. But it is now widely accepted that other animals experience genuine positive emotions, not simply the absence of negative emotions (Balcombe 2007; Boissy et al. 2007). This realization is important for two reasons. First, it is critical to welfare efforts on behalf of other animals (Boissy et al. 2007). Second, it brings to light the richness and shared psychology between humans and other animals (Balcombe 2007).

Emotions are ubiquitous in birds, as elsewhere in the animal kingdom (Bekoff 2005; Panksepp 2004). For instance, studies of emotional reactions to conspecific songs in white-throated sparrows (*Zonotrichia albicollis*) (Earp and Maney 2012), mood shifts in European starlings (*Sturnus vulgaris*) (Bateson and Matheson 2007), and fear responses in quail (*Coturnix coturnix*), (Mills and Faure 1986) provide evidence of both negative and positive avian emotions.

A review of the literature makes it clear that much more information is needed to understand chicken (and other bird) emotions. There are, however, a number of compelling findings implying that not only chickens experience emotions but that those emotions can be quite complex, given that they are combined with cognition and sociality.

### Fear responses

A host of studies provides convincing evidence of fear in chickens under a range of circumstances, including capture and restraint, open fields, and novelty. Chickens respond with a variety of complex behaviors adapted to each of the circumstances, e.g., tonic immobility upon restraint, and avoidance in some cases of the appearance of novel objects (Forkman et al. 2007, for a review of this literature). Emotional responses in chickens are accompanied by physiological reactions, i.e., tachycardia and bodily fever (also known as “emotional fever”), which underscore the shared characteristics of these emotions in chickens with other animals and humans (Cabanac and Aizawa 2000).

### Emotional response during anticipation

As discussed previously, one study of chickens tapped into time perception through an anticipatory emotional response. Laying hens were taught to discriminate three sounds which signaled either a positive (food reward), negative (a squirt from a water gun) or neutral (just waiting) outcome after a 15-s delay. The hens showed a range of emotional responses apparently in anticipation of the different future outcomes. For instance, in anticipation of the negative event, the birds showed more head movements and locomotion than in anticipation of both the neutral and positive event. The increased locomotion or stepping was consistent with pacing behavior, which is correlated with anxiety over an impending aversive encounter. In anticipation of the positive event, there was no increased stepping. Rather, the birds showed comfort behaviors (e.g., preening, wing flapping, feather ruffling, body scratching) consistent with relaxation (Zimmerman et al. 2011).

### Emotions and decision making

It is now well understood that humans and other animals make complex decisions based on emotions more than on facts, computations, or analyses (Bechara and Damasio 2005; Stephens 2008). In the case of many animals, complex foraging decisions appear to be made based upon emotional responses to various factors in the environment. The relationship between an emotional response to an environment and the decision to avoid or approach that environment, are key elements of animal welfare (Barnard 2007). Not surprisingly, chickens consistently choose to be in environments which offer better welfare as measured by several physiological welfare indicators (Nicol et al. 2009; Nicol et al. 2011a, b). In an investigation of the relationship between emotional response to three different environments and foraging decisions with risk trade-offs, Nicol et al. (2011a, b) found that laying hens had lower corticosterone levels (a physiological measure of stress) when making a positive environmental choice. Higher head temperature (another physiological marker of arousal) was also associated with preferred environments. Overall, the authors concluded: “Finding a link between a subset of physiological stress responses and decision making in a foraging context leaves open the possibility that birds may make use of emotional state variables as a proximate method of choosing between complex environments.” (p. 262).

### Emotions and cognitive bias

Cognitive bias is a deviation in judgment as a result of emotion-inducing experiences. It is tested (in humans and other animals) by exposing an individual to a positive or negative experience, and determining how those experiences shape perceptions of neutral or ambiguous stimuli (Mendl and Paul 2004). Depressed and anxious humans tend to interpret ambiguous situations more pessimistically than others (Mathews et al. 1995). Many nonhuman animals, including rats, dogs, primates, and starlings, show evidence of emotion-induced cognitive biases (Mendl et al. 2009, for a review of this literature). For instance, Bateson and Matheson (2007) found that European starlings who had recently been deprived of environmental enrichment in their home pens, flipped open the lids of food pots of an ambiguous color less often than did control birds. These results provide evidence that the birds’ negative mood was the basis for responding more pessimistically to ambiguous cues than individuals in a relatively more positive mood.

Wichman et al. (2012) examined the evidence for cognitive bias in hens housed in either basic or enriched pens. When they measured emotional responses and various measures of performance on a cognitive task, they found no differences in emotional state across the two treatments. Instead, differences between individuals were stronger than group differences. Individual factors such as fear level, relationship to their conspecifics, and motivation to feed were correlated with the birds’ behavior in the anticipation and cognitive bias tests. These results do not provide evidence of cognitive bias in chickens, but hint at the possibility that different manipulations, i.e., those that are stronger than individual differences, may reveal an effect.

### Emotional contagion and empathy

Emotions are often thought to be related to empathy. Empathy has been defined as having a similar emotional state to another as a result of the accurate perception of the other’s situation or predicament (Hatfield et al. 1993; Preston and de Waal 2002). Thus, there is both a cognitive and an emotional component to empathy. Emotions tend to influence more than one individual in a group, as they can be shared in a process known as emotional contagion. And, therefore, emotional contagion, an emotional response resulting in a similar emotion being aroused in an observer as a direct result of perceiving the same emotion in another, has been considered a simple form of empathy (De Waal 2003, 2008; Preston and De Waal 2002; Singer 2006). De Waal (2008) suggests that emotional contagion forms the basis of sympathetic concern (which involves some perspective-taking), and these lead to empathy-based altruism.

Emotional contagion, like other proximate psychological mechanisms, serves the ultimate purpose of providing a way for social animals to take in social cues about important circumstances and respond accordingly. Thus, emotional contagion has been demonstrated in many socially complex species such as dogs (*Canis lupus familiaris*) (Joly-Mascheroni et al. 2008), wolves (*Canis lupus*) (Romero et al. 2014), great apes (Anderson et al. 2004; Palagi et al. 2014), and pigs (Reimert et al. 2014). Although birds have not been traditional subjects in this area, recent work suggests a more sophisticated capacity for emotional response to conspecifics than previously realized, e.g., in ravens (*Corvus corax*) (Fraser and Bugnyar 2010) and geese (*Anser anser)* (Wascher et al. 2008).

In a study of how hens respond to their chicks’ distress, Edgar et al. (2011) found strong evidence for not only emotional contagion but also of empathy. Thirty-two hens experienced three conditions: a mildly aversive air puff into their cage (in order to provide experience with the aversive characteristics of an air puff), observation of an air puff into the cage where their chicks resided, or a control consisting of an air puff aimed outside of either cage. The hens were outfitted with heart rate monitors and were also monitored for eye and comb temperature with a thermal imaging camera. Hen behavior, vocalizations, and chick vocalizations were monitored continuously.

Importantly, the hens did not show any significant physiological or behavioral response to air puffs in their own cage. However, when they observed their chicks receiving the air puffs, there was a demonstrable response on the part of the mother hens, with physiological and behavioral changes indicating emotional distress. Their responses included increased heart rate and lower eye and comb temperatures (indicating vasoconstriction and increased body core temperature) as well as standing alert and maternal clucking. The hens’ responses were clearly reserved for when their chicks were experiencing the air puff, rather than a generalized negative response.

Interestingly, a later study showed that the hens’ responses were not simply due to increased vocalizations on the part of the chicks. The hens were responding to what they knew about the aversive nature of the air puff and the fact that it was being applied to their chicks. Their responses were mediated by a number of complex cues about whether the chicks were actually under threat, requiring them to integrate information coming in with their own knowledge of the stimulus in a potentially flexible and context-dependent way (Edgar et al. 2013). These findings not only provide evidence of emotional contagion in the hens but support the notion that hens are capable of a cognitively mediated empathic response. According to the authors: “We found that adult female birds possess at least one of the essential underpinning attributes of ‘empathy’; the ability to be affected by, and share, the emotional state of another” (http://bristol.ac.uk/news/2011/7525.html).

Follow-up studies examining the reactions of the chicks to the air puff show that the mother can act as a “social buffer” for the chicks, lessening their aversive reaction to the stimulus. However, there are individual differences across mother hens in their effectiveness as social buffers, with less emotional hens being better at buffering their chicks’ stress reaction (Edgar et al. 2015). These findings suggest that there are different “maternal styles” in mother hens which may be based upon differences in personality traits. Moreover, the social buffering observed in this study is not dissimilar to the phenomenon of “social referencing” in humans and other complex mammals, whereby the juvenile looks to the parent to determine how to respond emotionally to various situations. Chick reactions are less extreme when the hen’s responses are less extreme. Likewise, when a human child falls down, for instance, they immediately look to the mother to determine whether they should laugh or cry. The mother determines this by her own response, which is then modeled by the child. More relaxed parents tend to have more relaxed children over time (Walden and Ogan 1988). The similarity between social buffering and social referencing leaves open the possibility that they are connected through related cognitive-emotional-social capacities.

## Personality

Personality refers to “those characteristics of individuals that describe and account for temporally stable patterns of affect, cognition, and behavior” (Gosling 2008, p. 986). Or put another way, personality is a set of traits that differ across individuals and are consistent over time. The concept of personality is critically important for a complete understand of animals (including humans) as individuals. Instead of viewing other animals as one-dimensional, interchangeable units within a species, recognition of personality in other animals allows us to accurately see them as complex individuals with multi-dimensional characteristics. Furthermore, personality interacts with cognition and emotion, intimately shaping behavior and performance on a wide range of tasks.

Studies of personality in nonhuman animals have shown that personality traits are ubiquitous in the animal kingdom; a wide range of fish, birds, and mammals show persistent individual differences that can be organized along core personality dimensions, many of which overlap with those found in humans (Gosling 2008; Gosling and John 1999; Marino and Colvin 2015). Debate exists over the number and types of dimensions needed to characterize personality variation in most species of animals (Gosling 2008). In humans, there is broad agreement on a five-factor model of personality that includes the dimensions of openness, conscientiousness, extroversion, agreeableness, and neuroticism (McCrae and Costa 2008). Although some authors prefer to refer to *behavioral syndromes* or *temperament* in other animals (Reale et al. 2007), there is little distinction between these phenomena and personalities as observed and documented (Gosling 2008). With only slight variation of meaning, the different labels refer to the same category of phenomena. With that said, a number of avian species have demonstrated personality traits, e.g., zebra finches (*Taeniopygia guttata*) (David et al. 2011; Schuett et al. 2011), great tits (*Parus major*) (Groothuis Ton and Carere 2005), greylag geese (Kralj-Fiser et al. 2010) mountain chickadees (*Poecile gambeli*) (Fox et al. (2009), and Japanese quail, (*Coturnix japonica)* (Miller 2003; Miller et al. 2006), to name just a few.

There is an abundance of anecdotal evidence for individual personalities in chickens from sanctuaries, small farmers, and people who keep backyard chickens. And as should be clear from the previous section, mother hens show a range of individual maternal personality traits which appear to affect the behavior of their chicks.

Additionally, in studies examining the relationship between dominance status and personality traits in male chickens, three personality traits emerge—boldness, activity/exploration, and vigilance. In these studies, males are assessed for personality in various settings, such as a novel arena, and then placed together to determine how these factors impact the establishment of social status. Overall, the results demonstrate that when combatants are evenly matched in size, personality plays a role in the outcome of the challenge. Variation in several independent personality traits can influence the ability of an individual to obtain higher status. All three personality traits are positively correlated with higher social status (Favati et al. 2014a, b). Further work, at sanctuaries perhaps, focused on chicken personalities would clearly be of much interest to ethologists interested in chickens.

## Conclusions

In this paper, I have identified a wide range of scientifically documented examples of complex cognitive, emotional, communicative, and social behavior in domestic chickens which should be the focus of further study. These capacities are, compellingly, similar to what we see in other animals regarded as highly intelligent. They include:

1. Chickens possess a number of visual and spatial capacities, arguably dependent upon mental representation, such as some aspects of Stage four object permanence and illusory contours, on a par with other birds and mammals.
2. Chickens possess some understanding of numerosity and share some very basic arithmetic capacities with other animals.
3. Chickens can demonstrate self-control and self-assessment, and these capacities may indicate self-awareness.
4. Chickens communicate in complex ways, including through referential communication, which may depend upon some level of self-awareness and the ability to take the perspective of another animal. This capacity, if present in chickens, would be shared with other highly intelligent and social species, including primates.
5. Chickens have the capacity to reason and make logical inferences. For example, chickens are capable of simple forms of transitive inference, a capability that humans develop at approximately the age of seven.
6. Chickens perceive time intervals and may be able to anticipate future events.
7. Chickens are behaviorally sophisticated, discriminating among individuals, exhibiting Machiavellian-like social interactions, and learning socially in complex ways that are similar to humans.
8. Chickens have complex negative and positive emotions, as well as a shared psychology with humans and other ethologically complex animals. They exhibit emotional contagion and some evidence for empathy.
9. Chickens have distinct personalities, just like all animals who are cognitively, emotionally, and behaviorally complex individuals.

This is not to imply that the cognitive mechanisms underlying all of these apparent similarities are equivalent across species. Nor does it imply that higher-level explanations are always able to provide a thorough explanation of cognitive mechanisms. In fact, higher-level cognition is, unarguably, intertwined with more basic capacities and it may be contended that they are inseparable in many ways. Shettleworth (2010) argues that there is always an interplay between more fundamental cognitive mechanisms, e.g., associative learning, and other higher-level capacities, e.g., abstract thought, and that many human abilities derive from very basic cognitive processes. But the present findings do tell us that chickens, like other birds, are similar, in many ways, to mammals in their ethological complexity and that there are a number of findings that speak to the possibility of more complex capacities in chickens than heretofore recognized. These capacities serve as a list of promising areas of study for the future, as each needs to be explored further.

These findings come with a clear recommendation to continue our exploration of chickens’ ethological complexity within noninvasive, non-harmful, and more naturalistic contexts. A shift in how we ask questions about chicken psychology and behavior will, undoubtedly, lead to even more accurate and richer data and a more authentic understanding of who they really are.
